# CT-guided methylene blue staining to facilitate thoracoscopic resection of intermediate-to-deep pulmonary metastases in pediatric patients

**DOI:** 10.1007/s00383-026-06675-1

**Published:** 2026-10-12

**Authors:** Mikal Obed, Lucas Wennemann, Christin Linderkamp, Nathalie Pauer, Hoen-oh Shin, Jens Vogel-Claussen, Jens Dingemann, Rim Kiblawi

**Affiliations:** 1 Department of Pediatric and Adolescent Surgery, Hannover Medical School, Carl-Neuberg- Street 1, 30625 Hannover, Germany; 2 Department of Pediatric Oncology, Hannover Medical School, Carl-Neuberg-Street 1, 30625 Hannover, Germany; 3 Institute of Diagnostic and Interventional Radiology, Hannover Medical School, Carl- Neuberg-Street 1, 30625 Hannover, Germany; 4 Department of Radiology, Charité - Universitätsmedizin Berlin, Charitéplatz 1, 10117 Berlin, Germany

**Keywords:** CT-guided methylene blue staining, Computed tomography, Thoracoscopy, Pediatric pulmonary metastases

## Abstract

**Purpose:**

Thoracoscopic resection of pulmonary metastases offers a minimally-invasive approach but requires preoperative localization due to absent tactile feedback. CT-guided methylene blue staining (MBS) was implemented to facilitate resection of pulmonary metastases, including intermediate-to-deep lesions.

**Methods:**

We retrospectively analyzed pediatric patients (0–18 years) undergoing resection of pulmonary metastases (2007–2024). Lesion depth was classified as superficial (≤ 10 mm), intermediate (11–20 mm), or deep (> 20 mm). Primary endpoint was completeness of resection; secondary endpoints included perioperative and oncological outcomes. Subgroup analysis included patients with ≤ 2 pulmonary metastases.

**Results:**

Fifty-eight resections in 43 patients were included: 49 open, and 9 thoracoscopic following CT-guided MBS. Eleven lesions were stained prior to thoracoscopic resection: three superficial, seven intermediate, one deep lesion. All were resected with negative margins, without complications. Thoracoscopy following CT-guided MBS was associated with shorter operative times (65 vs. 157 min, *p* = 0.01), hospital stay (4.0 vs. 5.6 days, *p* = 0.01), and time-to-chemotherapy-resumption (2.25 vs. 18.6 days, *p* = 0.01). No differences were observed in pulmonary recurrence, disease-free interval, or six-month-survival.

**Conclusion:**

In pediatric patients with up to two pulmonary metastases, CT-guided methylene blue staining provided feasible preoperative localization, facilitating thoracoscopic wedge resection of superficial, intermediate and deep lesions without complications. Prospective multicenter studies should further evaluate efficacy and inclusion criteria.

## Introduction

Despite notable advancements in systemic therapy of pediatric malignancies, the management of pulmonary metastases (PM) in pediatric patients remains controversial. Outcomes for children and adolescents with PM have only shown minimal improvement over recent years [1, 2]. Metastases are detected synchronously with the primary tumor in up to one third of cases with solid tumors, with a further 15% − 20% of patients showing secondary recurrence at distant metastatic sites [3]. Among pediatric malignancies most commonly associated with PM are Wilms tumor, hepatoblastoma, rhabdomyosarcoma, soft tissue sarcomas, Ewing’s sarcoma, and osteosarcoma [4]. Although metastatic dissemination can occur at various anatomical sites, including lymph nodes, liver, bone, and brain; the majority are observed in the lungs [2].

Although the definite diagnosis is established histologically, computed tomography (CT) scan is widely used for detection and characterization of PM as part of oncological staging [5–7]. Advances in thin-slice CT have further improved spatial resolution, enabling detection of pulmonary lesions with a sensitivity approaching that of surgical bimanual palpation [8, 9].

When PM persist after initial chemotherapy, complete surgical resection may be considered as part of multimodal treatment and has been associated with improved survival in selected pediatric patients [4, 10–12]. Wedge resection with negative margins is generally sufficient to achieve local control while preserving healthy lung parenchyma [13–15]. Bimanual palpation through thoracotomy or sternotomy remains the standard approach for identifying and resecting PM, allowing detection of lesions that may not be apparent on imaging alone [16]. However, non-calcified or deeply located intraparenchymal lesions may be difficult to detect by palpation. Moreover, open approaches—including unilateral or bilateral thoracotomy, clamshell thoracotomy, and sternotomy—are associated with considerable morbidity [12, 14, 17].

Thoracoscopic resection has therefore gained increasing importance as a minimally invasive option, offering faster recovery, reduced morbidity, and favorable perioperative outcomes [18–20]. However, the absence of tactile feedback during thoracoscopy poses a particular challenge for lesions that are not directly visible, making accurate preoperative localization essential.

There is a paucity of robust data on thoracoscopic resection of pediatric PM following preoperative lesion localization [21]. Several localization techniques have been described in pediatric patients; however, data specifically evaluating CT-guided methylene blue staining (MBS) for thoracoscopic resection of intermediate-to-deep pulmonary lesions remain limited. At our institution, CT-guided MBS has been used since 2021 to localize CT-detectable pulmonary lesions in patients with no more than two PM, who were clinically stable enough to undergo thoracoscopic surgery.

The aim of this study was to evaluate the technical feasibility, perioperative and early oncological outcomes of thoracoscopic resection following preoperative CT-guided MBS in selected pediatric patients with ≤ 2 PM, including pulmonary lesions of various intraparenchymal depths.

## Materials and methods

### Study design and patient population

Ethical approval was obtained prior to study initiation (Approval No. 11696-BO-K-2025). We retrospectively analyzed clinical data from pediatric patients (0–18 years) diagnosed with solid tumors and pulmonary metastases who underwent PM resection at our center between January 2007 and December 2024. Oncologic treatment adhered to the guidelines of the German Society for Pediatric Oncology and Hematology (GPOH), and pre- and post-operative radiological findings were routinely reviewed in interdisciplinary pediatric tumor board meetings. Prior to 2021, surgical resection was performed exclusively via open thoracotomy (posterolateral thoracotomy, sternotomy, or clamshell thoracotomy) combined with bimanual lung palpation. Since 2021, a thoracoscopic approach preceded by CT-guided MBS has been implemented for hemodynamically stable patients presenting with ≤ 2 PM per hemithorax and an adequate puncture window on pediatric chest CT. To address potential confounding factors related to polymetastatic disease extent, a focused comparative subgroup analysis was conducted exclusively comprising patients with ≤ 2 PM per hemithorax across both surgical arms.

### Preoperative evaluation and lesion depth classification

All patients underwent high-resolution chest CT prior to surgery. Consistent with established criteria in adult series and pediatric literature, PM depth from the visceral pleural surface was categorized on preoperative CT as superficial (≤ 10 mm), intermediate (> 10–20 mm), or deep (> 20 mm) [22, 23].

### Preoperative CT-guided methylene blue staining (MBS)

Localization was performed under general anesthesia, aiming for an intervention-to-incision interval of < 1 h to prevent stain fading. Following written parental informed consent, CT scan acquisition was performed in the prone or supine position (Somatom X.cite; Siemens Healthineers, Forchheim, Germany). The access pathway was collaboratively determined by the interventional radiologist and the operating surgeon, to enhance the intraoperative visualization of the staining.

**Fig. 1:**
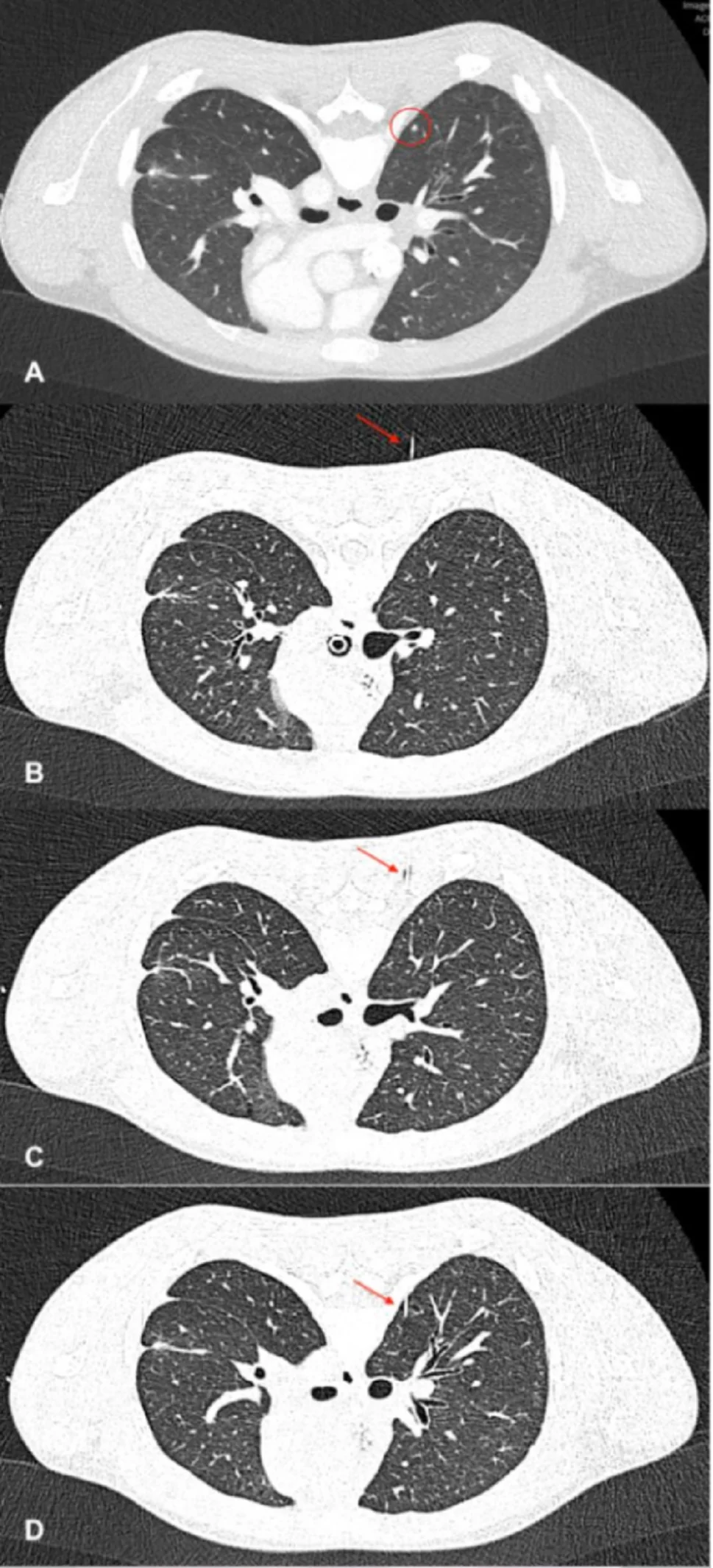
CT-guided methylene blue staining prior to thoracoscopy. The arrow indicates the needle trajectory **A** CT-guided localization of suspected lung metastasis in the right upper lobe **B**–**D** Transthoracic CT-guided methylene blue staining of suspect nodule

Under sterile conditions and intermittent CT guidance, a 21-gauge needle was advanced toward the target lesion, with precise documentation of needle trajectory and tip positioning relative to the nodule (Fig. 1). Methylene blue solution was prepared by diluting 1 mL of dye in 4 mL of 0.9% sodium chloride solution. Depending on nodule location and depth, 1 to 3 mL of the solution was administered, with larger volumes utilized for deeper lesions. Upon needle retraction, the puncture channel and visceral pleura were stained. Post-procedural CT imaging, supplemented by 3D volume-rendering reconstructions when required, evaluated dye distribution and its spatial relationship to the target lesion. These localization parameters were communicated directly to the operating surgeon to guide the direction and extent of the subsequent wedge resection.

### Surgical techniques

Open surgical resection was carried out through posterolateral thoracotomy, sternotomy, or clamshell thoracotomy. Bimanual lung palpation was systematically performed by a team of three (posterolateral thoracotomy) or four surgeons (sternotomy or clamshell thoracotomy), including at least two attending consultants.

Thoracoscopic wedge resection encompassed the stained pleural and parenchymal area; the extent of parenchymal excision was determined intraoperatively based on stain distribution and surgical assessment. Endoscopic linear staplers equipped with vascular cartridges were applied through macroscopically unstained, healthy-appearing lung parenchyma to ensure airtight closure and bronchovascular sealing while preventing inclusion of the target lesion within the staple line. Upon retrieval, specimens were inspected and palpated intraoperatively to confirm complete lesion inclusion and evaluate resection margins.

### Endpoints, histopathology, and postoperative management

The primary endpoint was complete resection of PM. Secondary endpoints encompassed hospital length of stay, time to chemotherapy resumption, postoperative complications, pulmonary recurrence and patient survival outcomes. Resection status was histopathologically evaluated based on completeness of resection, metastatic burden in the specimen, and staining results from preoperative MBS. Only complete histological resection (R0) was considered as successful resection of the PM. Follow-up chest CT was performed 4 weeks to 3 months postoperatively. Thoracic drain removal followed a standardized institutional protocol: clear fluid drainage < 50 mL/24 h in children > 2 years (< 20 mL/24 h in children ≤ 2 years), absence of air leak or hemothorax, and radiographic evidence of complete lung re-expansion. Systemic chemotherapy was resumed following adequate surgical recovery and wound healing, as determined jointly by the treating pediatric oncology team and oncologic surgeon according to treatment protocol guidelines.

### Statistics

Qualitative variables were described as percentages, while quantitative variables were expressed as means and standard deviations. Comparative analyses were conducted using the Fisher’s exact test for categorical variables and Welch’s t-test for continuous variables. P-value < 0.05 was considered significant. Statistical analyses were performed using Microsoft Excel 2010, Microsoft Corp. (Redmond, Washington, United States) and Statistical Analysis System (SAS) Software Version 9.4, SAS Institute Inc. (Cary, United States), and Jamovi Version 2.3.28.

## Results

**Fig. 2:**
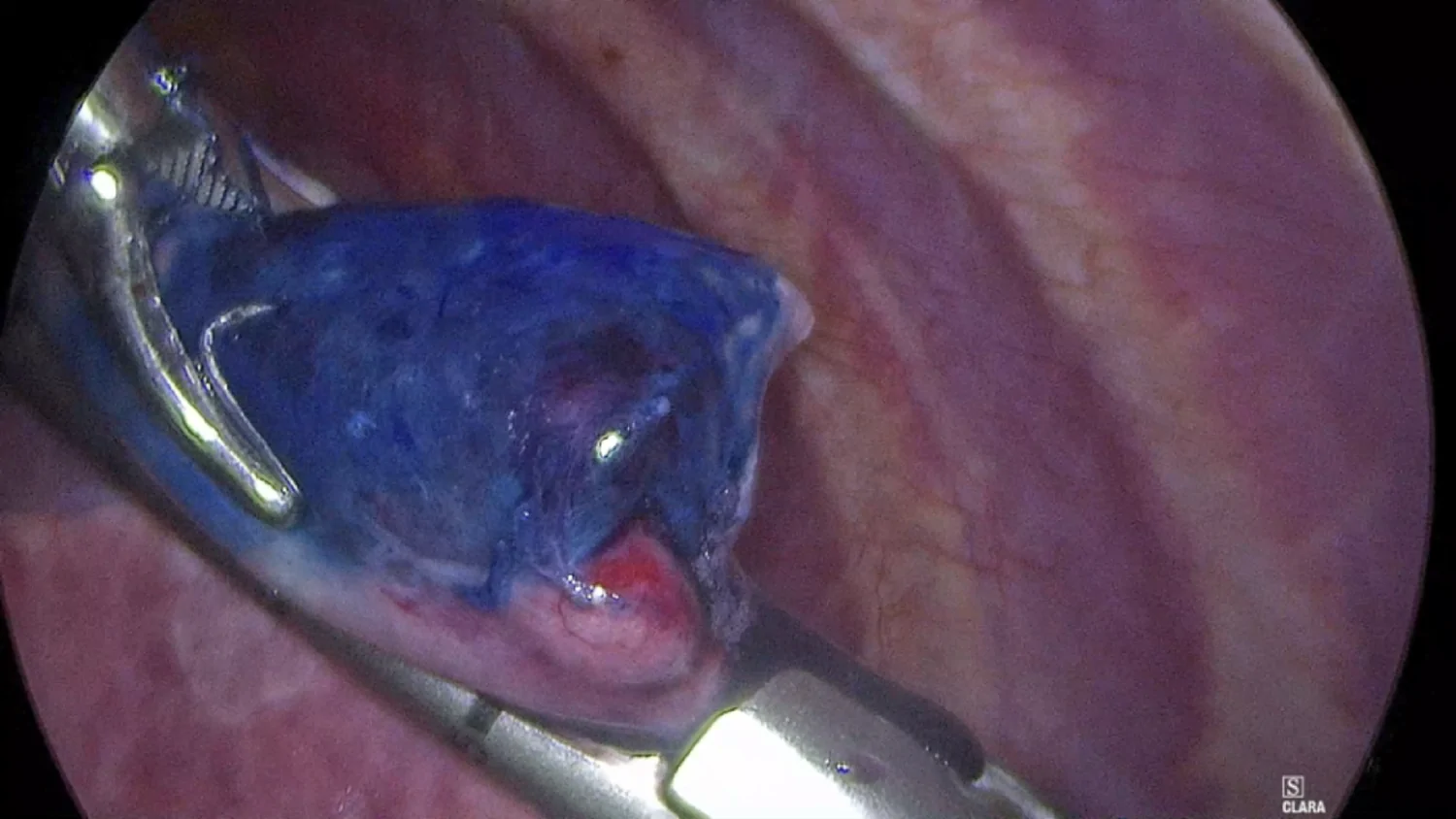
Intraoperative identification of suspicious nodule after CT-guided staining with methylene blue. The puncture channel and pleura have been stained to ensure intraoperative identification

A total of 58 pulmonary metastasis resections in 43 patients were identified and included in this study. Of these, 49 resections were performed via open surgery; nine by thoracoscopy with prior CT-guided MBS to target a total of 11 lesions. The mean age of patients who underwent CT-guided MBS was 14 years, with the majority being male (78%) (Table 1). The diagnoses encompassed a diverse range of tumor entities (Table 3). All 11 nodules were successfully stained under CT guidance (technical success rate, 100%) (Fig. 2). Among thoracoscopically resected nodules, three were classified as superficial (≤ 10 mm from the pleural surface), seven as intermediate (11–20 mm), and one as deep (> 20 mm) (Table 2). In one case, staining faded during the interval between CT-guided marking and thoracoscopy, resulting in diffuse stain distribution. Nevertheless, the area of maximal staining allowed identification of the target region and margin-negative resection was achieved. Histopathological examination confirmed R0 resection in all thoracoscopically resected lesions. Notably, in one case, a deeply located recurrent nodule in the right upper lobe—previously undetectable by palpation during open surgery—was successfully resected with clear margins using MBS (Table 1, patient 3). No complications were encountered during the CT-guided staining procedure.

**Table 1:** Demographics of all patients that received CT-guided MBS and subsequent thoracoscopy

Patient no	Gender	Age at surgery (y)	Diagnosis	Suspected lesion	Localization	AC	Re-Operation*	6-month survival
1	F	11	Osteosarcoma	1	L LL	N	N	Y
2	M	16	Synovial carcinoma	1	L UL	Y	Y	Y
3	M	15	Osteosarcoma	1	R UL	Y	Y	Y
4	M	16	Ewing’s sarcoma	2	L UL, L LL	Y	N	Y
5	M	17	Ewing’s sarcoma	1	L UL	N	N	Y
6	M	16	Germ cell tumor	2	L LL	N	N	Y
7	M	6	Hepatoblastoma	1	L UL	Y	N	Y
8	M	17	Lymphoma	1	L UL	N	N	Y
9	F	13	Ewing’s sarcoma	1	R LL	Y	N	Y

* M* male, *F* female, *y* years, *AC* Adjuvant chemotherapy, *Y* Yes, *N* No, *L* Left, *R* Right,* LL* lower lobe, *UL* upper lobe

* A secondary pulmonary resection was indicated due to pulmonary recurrence

**Table 2:** Surgical data from patients after thoracoscopy with prior CT-guided MBS

Patient	Number of PM	Depth of nodule from pleural surface*	Depth of nodule from pleural surface (mm)	Size of nodule (maximum diameter, mm)	Surgery duration (min)	PR	Time until PR (months)	Length of stay (d)
1	1	IM	11	4	45	N	N/A	3
2	1	S	6	6	38	Y	15	9
3	1	D	24	12	99	N	N/A	6
4	1	IM IM	13 15	5 2	51	Y	17	3
5	1	IM	16	17	42	Y	5	2
6	2	IM S	18 8	9 8	141	N	N/A	2
7	1	IM	14	5	59	N	N/A	2
8	1	IM	19	9	65	N	N/A	4
9	1	S	8	8	42	N	N/A	4

* PM* Pulmonary metastases, *PR* Pulmonary recurrence, *S* Superficial, I*M* intermediate, *D* Deep

*Superficial ≤ 10 mm

*Intermediate 11–20 mm

*Deep > 20 mm

### Thoracoscopic Resection after CT-guided MBS

Mean time for thoracoscopic surgery was 65 ± 35 min (38–141 min) (Table 2). Histology confirmed margin-negative R0-resection in 100% of cases following thoracoscopy with prior CT-guided MBS. No complications related to thoracoscopy were observed. The mean length of hospital stay was 4 days. At six-month follow-up, all patients who underwent thoracoscopic resection with CT-guided MBS survived. Pulmonary recurrence was observed in three patients, at 5, 15 and 17 months after surgery.

### Comparative analysis of thoracoscopy with prior MBS vs. open surgery

Compared with open surgery, thoracoscopy following MBS was associated with shorter mean operative time (65 vs. 157 min, *p* = 0.01), shorter mean hospital stay (4.0 vs. 5.6 days, *p* = 0.01), and shorter mean time from surgery to resumption of chemotherapy (2.25 vs. 18.6 days, *p* = 0.01) (Table 3). No complications were observed following thoracoscopic surgery, compared with four postoperative pleural effusions, one pneumothorax, and two wound dehiscences with infection (one of which necessitated antibiotic therapy) in the open surgery group. The difference in postoperative complications was not statistically significant (*p* = 0.23). After open surgery, four patients (11%) required a patient-controlled analgesia pump, whereas all patients following thoracoscopy were managed with standard analgesia. No significant difference was observed in achieving histopathological R0 resection between thoracoscopic and open surgery (*p* = 0.09). Pulmonary recurrence occurred in 40% of patients after thoracoscopy and 44.7% after open surgery (*p* = 0.79). There was no significant difference in time to pulmonary recurrence (*p* = 0.3) or six-month survival (*p* = 0.5).

**Table 3:** Comparison between open surgery and thoracoscopy with prior CT-guided MBS.

	Open surgery	VATS with prior MBS	*P-*value
Number of Surgeries	49	9	
Postoperative initiation of chemotherapy (days)	18.6	2.25	0.01*
Operation Time (minutes)	157	65	0.01*
Length Of Hospital Stay (days)	5.6	4	0.01*
Time to Pulmonary Recurrence (months^#^)	7.7	11	0.3

* Statistical significance was considered at a p-value of < 0.05

# Postoperative months after surgery

### Comparative analysis of thoracoscopy with prior MBS vs. open surgery in patients with ≤ 2 PM

Given that metastatic burden may influence the choice of surgical approach, a secondary analysis was performed with patients with unilateral pulmonary involvement and ≤ 2 PM in both groups (Table 4). Significantly shorter operative times were observed for thoracoscopic surgery compared to open surgery (*p* = 0.01), along with shorter time to chemotherapy (*p* = 0.01) and shorter length of hospital stay (*p* = 0.04). No differences were found regarding the occurrence of pulmonary recurrence in both groups (*p* = 0.3). Furthermore, no differences in time to pulmonary recurrence and six-month survival were observed (*p* = 0.3).

**Table 4:** Comparison between open surgery and thoracoscopy with prior CT-guided MBS in oligometastatic disease (pulmonary metastases ≤ 2).

	Open surgery	VATS with prior MBS	P-value
Patients, n	22	9	-
Surgical procedures, n	27	9	-
Age at time of surgery (mean; years)	12.5	14.9	-
Male, n (%)	13 (59%)	7 (77%)	-
Calcifying tumors, n (%)	10/22 (45%)	5/9 (55%)	-
Operation time (mean, minutes)	109.8	65	0.01*
Duration of chest tube (days)	3.0	1.25	0.09
Complications, n (%)	7/27 (26%)	0/9 (0%)	0.14
Length of hospital stay (days)	5.8	3.3	0.04*
Postoperative time to chemotherapy (days)	18.6	2.3	0.01*
Recurrence at 6 months, n (%)	3/27 (11%)	0/9 (0%)	0.3
Survival at 6 months, n (%)	26/27 (96%)	9/9 (100%)	0.3

* Statistical significance was considered at a p-value of < 0.05

## Discussion

This study provides an evaluation of CT-guided MBS prior to thoracoscopic resection of selected PM in children and adolescents. Our findings demonstrate a 100% technical success rate in lesion marking without procedure-related complications. Complete histological resection (R0) was confirmed in all patients with ≤ 2 PM.

While most available evidence regarding CT-guided MBS derives from adult cohorts, pediatric data remain scarce and are largely restricted to superficial or subpleural nodules [21]. In pediatric practice, thoracoscopic resection of intermediate-to-deep PM often necessitates multiple concurrent localization modalities [24]. In our cohort, 8 of 11 nodules were located more than 10 mm from the pleural surface, including seven at intermediate depth (11–20 mm) and one deeply intraparenchymal lesion (> 20 mm), all of which were successfully localized using CT-guided MBS as single technique. Thus, MBS may facilitate localization of selected pediatric PM beyond the superficial subpleural compartment, although the small sample size and retrospective design preclude conclusions regarding comparative effectiveness.

Alternative marking techniques utilized prior to pediatric thoracoscopy, including lipiodol, hookwire, radiotracers, intraoperative ultrasound, and indocyanine green (ICG), carry technical limitations. Lipiodol may induce pleural adhesions, while hookwire risks dislocation and complications such as pneumothorax or hemorrhage [25, 26]. Comparative studies suggest that MBS achieves equivalent intraoperative lesion identification to hookwire while reducing procedure time, hospital stay, and complication rates [27, 28, 31]. However, these findings derive from different study populations and techniques and cannot be directly extrapolated to the present cohort. Radiotracer-based localization (technetium-99 m macroaggregates) is largely limited to sub-centimeter, superficial lesions and requires extensive logistics, including nuclear medicine coordination [29]. In our cohort, 8/11 lesions (73%) were located more than 10 mm from the pleural surface, limiting the applicability of this approach. Intraoperative percutaneous ultrasound is constrained by probe size and aerated lung interference, and is primarily applicable to peripheral, subpleural lesions, with evidence limited to adult cohorts [30]. ICG demonstrates efficacy comparable to MBS and wire-guided techniques, but is limited by shallow tissue penetration (≤ 10 mm) and the requirement for near-infrared imaging, which depends on center-specific resources [31]. In contrast, MBS can be identified under standard white light without specialized intraoperative imaging equipment, and our series suggests feasibility not only for superficial but also for intermediate-to-deep PM.

A recognized technical limitation of liquid staining, including methylene blue, is premature diffusion or washout of the stain when surgery is delayed [32]. To optimize visual localization, we restricted the interval between CT-guided MBS and skin incision to less than one hour. Although one lesion exhibited diffused staining at the time of surgery, the area of maximal staining could be identified, allowing complete resection. This observation underscores the importance of minimizing the interval between localization and resection, whilst carefully interpreting the intraoperative distribution of the stain.

In the present cohort, thoracoscopic resection following MBS was associated with shorter operative time, shorter hospital stays, and earlier resumption of chemotherapy compared with open resection. However, these comparisons must be interpreted with caution, as the observed differences cannot be attributed directly to CT-guided MBS or the thoracoscopic approach. Patients undergoing CT-guided MBS represented a selected population with limited disease and lesions that were anatomically suitable for percutaneous CT-guided staining and thoracoscopic wedge resection. Open thoracotomy combined with bimanual palpation remains the standard approach for patients with multiple metastases and may be warranted for select tumor types (e.g., osteosarcoma). The present findings should therefore not be extrapolated to patients with polymetastatic disease or lesions in locations unfavorable for thoracoscopic resection. Within this selected setting, CT-guided MBS represents one possible approach to preoperative localization of CT-identifiable PM considered suitable for thoracoscopic resection.

Despite these encouraging results, several limitations should be acknowledged. The retrospective, single-center design limits the generalizability of the findings. While restricting comparative subgroup analysis to patients with ≤ 2 PM reduced differences in metastatic burden between groups, the small MBS cohort did not eliminate the potential for selection bias or residual confounding. In addition, heterogeneity in tumor type, disease stage, and comorbidities may have influenced perioperative outcomes. The efficacy of MBS is also inherently dependent on CT detectability, as lesions that cannot be identified on imaging cannot be interventionally localized. Finally, long-term oncological outcomes remain unknown, and the small sample size limits the assessment of uncommon complications and meaningful differences in oncological outcomes between approaches.

Overall, these findings suggest that CT-guided MBS, an established localization technique for PM in adults, may also be applicable to intermediate-to-deep lesions in selected pediatric patients undergoing thoracoscopic resection.

## Conclusion

In pediatric patients with up to two pulmonary metastases, CT-guided methylene blue staining provided technically feasible preoperative localization and facilitated thoracoscopic wedge resection, including for intermediate-to-deep lesions. No procedure-related complications occurred. Prospective multicenter studies are warranted to further evaluate its efficacy and define standardized patient-selection criteria.

## Data Availability

The datasets generated and/or analyzed during the current study are not publicly available due to data protection and privacy regulations but are available from the corresponding author on reasonable request and subject to institutional approval.
